# A Crossover Study of Virtual Reality Exposure for Emotional and Physiological Regulation in Mild Dementia

**DOI:** 10.3390/brainsci15050470

**Published:** 2025-04-28

**Authors:** Paula Latorre, Cleiton Pons Ferreira, Francisco Nieto-Escamez

**Affiliations:** 1 CIBIS Research Center (Centro de Investigación para el Bienestar y la Inclusión Social), University of Almeria, 04120 Almería, Spain; plr791@ual.es; 2 Department of Psychology, Campus La Cañada, University of Almeria, Ctra. Sacramento S/N, 04120 Almería, Spain; 3 Instituto Federal de Educação, Ciência e Tecnologia do Rio Grande do Sul, Rio Grande 96201-460, Brazil; cleiton.ferreira@riogrande.ifrs.edu.br

**Keywords:** dementia, immersive virtual reality, non-pharmacological interventions, psychological well-being, personalized content, heart rate

## Abstract

(1) Background: Immersive virtual reality (IVR) has emerged as a promising non-pharmacological intervention to promote relaxation and improve emotional well-being in this population. (2) Methods: This crossover study evaluated the effects of IVR on anxiety and psychological well-being in a sample of eight participants with mild dementia attending a day-care center. Participants underwent two conditions: an experimental condition involving relaxing nature-based VR scenarios (Nature Treks VR) and a control condition using personalized YouTube videos on a tablet. Each condition lasted 12 sessions. Assessments included heart rate (HR), the I-PANAS-SF, the reduced State–Trait Anxiety Inventory (STAI-r), behavioral observations, and a subjective response questionnaire. (3) Results: A significant reduction in HR over time was found during IVR exposure, suggesting a calming physiological effect not observed in the control condition. While changes in PANAS and STAI-r scores were not statistically significant, the PANAS score improvement in the experimental condition approached statistical significance (*p* = 0.054) and was just below the minimal clinically important difference (MCID), suggesting a potentially meaningful trend. Behavioral responses were higher during YouTube sessions, likely due to personalized content. All participants rated the IVR experience positively on the subjective questionnaire, indicating high acceptability, though social desirability bias cannot be excluded. (4) Conclusions: IVR appears to be a feasible and acceptable intervention for individuals with dementia, warranting further investigation.

## 1. Introduction

Dementia is a global health challenge, affecting an estimated 57 million people worldwide [1]. Among the numerous psychological comorbidities associated with dementia, generalized anxiety disorder is particularly prevalent, with studies reporting a prevalence of up to 38% in this population, especially among individuals with mild cognitive impairment [2]. Anxiety in dementia patients not only exacerbates cognitive and functional decline but also significantly diminishes their quality of life, creating a pressing need for effective interventions [3]. Traditionally, pharmacological approaches have been the cornerstone of anxiety management in this population. However, these treatments often come with limitations, including adverse side effects, potential acceleration of cognitive decline, and limited emotional benefits. Consequently, there is a growing interest in exploring non-pharmacological strategies that can complement or even replace traditional methods, offering safer and more holistic alternatives.

Among the emerging non-pharmacological interventions, immersive virtual reality (IVR) has gained attention as a promising tool for mental health applications [4]. IVR enables users to engage with personalized virtual environments that simultaneously stimulate multiple senses, creating immersive experiences that can evoke relaxation, reduce stress, and promote positive emotional states. This technology offers a unique opportunity to address anxiety in individuals with mild dementia or early-stage Alzheimer′s disease, providing tailored experiences that minimize risks while maximizing emotional and psychological benefits. Moreover, IVR can be adapted to the specific needs and preferences of each patient, making it a highly individualized intervention.

Recently, a scoping review by Buele et al. [5] emphasized the emotional and user experience outcomes of VR in neurodegenerative disorders, noting improvements in apathy, mood, and quality of life—particularly in immersive systems designed with user adaptability in mind. These results suggest that IVR not only engages users effectively but may also yield psychophysiological benefits relevant to anxiety management.

In parallel, recent studies have increasingly examined the emotional benefits of nature-based virtual environments for individuals with dementia. Kim et al. demonstrated that immersive exposure to natural scenery in virtual reality can help reduce physiological stress and promote emotional relaxation in this population, underscoring the value of naturalistic virtual stimuli in dementia care [6,7]. Furthermore, a pilot study by Soares et al. [8] explored the use of 360° videos of personally meaningful locations during reminiscence therapy with individuals living with dementia. Although no statistically significant differences were found between the immersive and non-immersive conditions in terms of anxiety and depression reduction, the approach demonstrated feasibility and acceptability, supporting the continued exploration of personalized immersive content.

Building on previous findings, our study explores the use of IVR as a non-pharmacological intervention to reduce anxiety and improve psychological well-being in individuals with mild dementia. Specifically, we investigate the effects of exposure to relaxing virtual environments, such as nature-based scenarios (e.g., Nature Treks VR) and 360-degree videos of personally significant places or activities. To evaluate the impact of IVR, we employ a multimodal assessment approach, including physiological measures (e.g., heart rate), standardized psychological questionnaires (e.g., STAI-r for anxiety and I-PANAS-SF for psychological well-being), observational records during IVR sessions, and subjective feedback from participants. Our hypothesis is that IVR-based interventions will significantly reduce anxiety levels and enhance emotional well-being in this population.

By examining the potential of IVR as a therapeutic tool, this study aims to contribute to the growing body of evidence on non-pharmacological interventions for dementia-related anxiety. Furthermore, we seek to determine whether IVR can be integrated into routine care practices to improve the quality of life and emotional resilience of individuals living with dementia.

## 2. Materials and Methods

### 2.1. Participants

The study included ten Spanish nationals recruited from the Early Intervention Center for Alzheimers and other Neurological Disorders, affiliated with the Almerian Federation of Associations for People with Disabilities (FAAM) in Almería, Spain. Following a review of medical records, two participants were excluded due to a history of vertigo or epileptic episodes, resulting in a final sample of eight individuals (four women and four men). Participants ranged in age from 75 to 89 years and were assessed using two standardized clinical tools: the Clinical Dementia Rating (CDR) scale and the Global Deterioration Scale (GDS) by Reisberg. Based on these assessments, participants exhibited cognitive impairment levels ranging from “questionable dementia” to “moderate dementia” (CDR scores) and stages 3 to 5 on the GDS, indicating mild to moderate cognitive decline (see Table 1).

Exclusion criteria included a history of seizures, vertigo, epilepsy, hallucinations, severe behavioral disorders (e.g., high aggression), or recurrent absences from the center that could interfere with participation. Prior to enrollment, both participants and their family members were provided with detailed information about the study’s objectives, procedures, and legal responsibilities. Written informed consent was obtained from all participants or their legal representatives. No monetary compensation was provided for participation.

### 2.2. Design

An experimental crossover design was employed with two conditions: experimental and control. Participants were randomly assigned to both conditions. The experimental condition involved immersive virtual reality (IVR) sessions combining Nature Treks VR—a software application featuring naturalistic virtual environments—and 360° YouTube videos tailored to participants’ personal interests. The control condition consisted of standard cognitive stimulation activities routinely implemented in the care center, including an object-matching game and a word search task delivered via tablet applications.

### 2.3. Instruments

#### 2.3.1. Neuropsychological, Attitude, and Behavioral Questionnaires

To assess the psychological aspects and level of dementia in participants, the following tools were used: the Clinical Dementia Rating (CDR) scale [9], the Global Deterioration Scale (GDS) [10], the abbreviated and Spanish-adapted version of the Positive and Negative Affect Schedule (PANAS) (I-PANAS-SF) developed by Gargurevich [11], and the short version of the State–Trait Anxiety Inventory (STAIr) developed by Fernández-Blázquez et al. [12] (see Table 2). The Clinical Dementia Rating (CDR) scale evaluates six domains: memory, orientation, judgment and problem-solving, home and hobbies functioning, personal care, and social life. The evaluation of these areas is classified into five levels, with 0 indicating the absence of dementia and 3 indicating severe dementia.

The Global Deterioration Scale (GDS) consists of seven possible levels. Levels 1 to 3 indicate pre-dementia or cognitive decline, while levels 4 to 7 indicate varying stages of dementia.

The I-PANAS-SF is a short version of the PANAS that uses 10 of the 20 items from the original scale, measuring positive (5 items) and negative (5 items) affect of the participants. In this case, the Spanish-adapted version was used [11].

The STAIr is a reduced version of the STAI, which, like the original scale, captures two components of anxiety: state and trait. The “state” component refers to the temporary emotional state (corresponding to items 27, 29, 30, 31, 32, 33, and 38 of the STAI), while the “trait” component refers to a constant personality tendency (corresponding to items 1, 5, 8, 12, 18, and 19 of the STAI).

An ad hoc behavioral record was created to gather data on observable aspects of the participants during exposure to virtual environments. This record includes information on posture, facial expressions, verbalizations, and reminiscence, with four possible scores: “Only at the beginning”, “Once during the experience”, “At least twice during the experience”, and “Most of the time”.

Additionally, a subjective response questionnaire was developed to assess how participants felt during the activity. This questionnaire consisted of 5 closed-ended questions with dichotomous answers (Yes/No): “Do virtual scenarios excite you?”, “Do virtual scenarios calm you?”, “Do virtual scenarios make you feel upset?”, “Do virtual scenarios make you feel anxious?”, and “Do virtual scenarios seem realistic to you?”.

#### 2.3.2. Hardware and Software

To record the physiological response of the participants (heart rate), eight smart wristbands Xiaomi Band 7 (Xiaomi Corporation, Beijing, China) were used.

The Nature Treks VR v. 1.29 software (Greener Games, Telford, UK) is an application with different natural virtual environments categorized by themes. The aim of this application is to generate a state of relaxation through immersion in a natural environment using VR. For the intervention, all virtual scenarios offered by the software were used: Red Savanna, Red Fall, Green Meadows, Blue Ocean, White Winter, Black Beginning, and Blue Deep.

A set of three Oculus Go 64 GB headsets (Oculus VR, Menlo Park, CA, USA) were used to display the virtual environments. These headsets are equipped with an LCD (2560 × 1440 pixels), a Qualcomm Snapdragon 821 microprocessor, an Adreno 530 graphic card, and a refresh rate of 72 Hz.

For the control condition, cognitive stimulation activities were delivered via a 3GO GT10K2IPS tablet (3GO, San Francisco, CA, USA). Two applications were used: (1) ”Oya: Juego de Alzheimer, Emparejar”, an object-matching game (Akin Türker İlkyaz, İzmir, Turkey), and (2) ”Sopa de letras Español”, a word-search puzzle (Playvalve S.L., Barcelona, Spain).

### 2.4. Procedure

**Figure 1:**
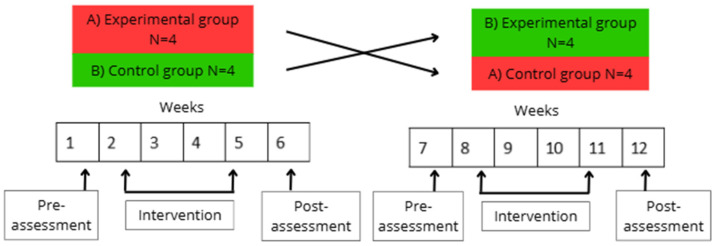
Study timeline. The figure displays the sequence of sessions, assessments, and interventions administered throughout the study. Participants underwent two phases: the experimental condition (immersive virtual reality) and the control condition (tablet-based activities). Each phase consisted of 12 sessions, with pre- and post-intervention assessments conducted for both conditions. The crossover structure allowed all participants to experience both interventions in alternating phases, ensuring intra-individual comparison and control for order effects.

The study lasted for 12 weeks and took place at the center where the participants attended daily. It was divided into two phases: the first phase consisted of an experimental group and a control group (EG and CG), and the second phase involved swapping the participants between each group, so that the experimental group became the control group, and vice versa. To minimize potential carryover effects between phases, a washout period was implemented following the conclusion of the first phase. The second phase began three weeks after the end of the first, allowing sufficient time for physiological and emotional states to stabilize. Each phase lasted five weeks and was divided into three stages. (1) An initial neuropsychological (GDS and CDR) and psychological (I-PANAS-SF and STAIr) assessments that took one week. (2) An intervention procedure carried out three times a week for four weeks, resulting in a total of twelve virtual reality exposure sessions. The structure followed during the intervention stage was as follows: During the first and third weeks, participants were presented with natural environments in virtual reality, randomly selected from the Nature Treks software. During the second and fourth weeks, participants watched 360° videos downloaded from YouTube based on their interests and requests. Thus, of the twelve intervention sessions, half of them involved viewing pre-set environments from Nature Treks, and the other half involved virtual environments based on the participants’ preferences. The sequence of interventions, session structure, and timing of assessments are detailed in Figure 1. Every session, when participants arrived at the center, smartwatches were placed on them to record heart rate during the activity. Around 10 a.m., participants (both EG and CG groups) were moved to a room and seated in comfortable armchairs. The experimental group wore the VR headsets, and the virtual environments were presented, while the control group was offered an alternative activity, in this case, cognitive stimulation exercises on a tablet. Each session lasted 15 min for both groups. Physiological measurements for all participants were recorded at five different times: one pre-measurement upon arrival in the room after sitting in the armchairs, three measurements during the activity at 5, 10, and 15 min after starting, and one final measurement after the activity, once the VR headsets (EG) or the tablet (CG) were removed. Additionally, during the session, the evaluator filled out the behavior questionnaire for each participant in the EG. Participants in the EG were then assessed with the subjective questionnaire using a dichotomous response format. (3) The intervention concluded with a post-assessment of the psychological variables (I-PANAS-SF and STAIr), following the same protocol as the pre-assessment. Post-intervention assessments of psychological variables (I-PANAS-SF and STAI-r) were conducted during the week following the final intervention session of each phase—specifically, in weeks 6 and 12 of the study. This timing was chosen to evaluate the short-term effects of the interventions beyond the immediate session.

### 2.5. Analysis

Quantitative data were analyzed by using the software JASP 0.16.4.0. A repeated measures ANOVA was used to compare pre- and post-intervention scores obtained in psychological assessments with STAIr (pre- vs. post-scores) and I-PANAS-SF (pre- vs. post-scores).

A repeated measures ANOVA was also used to test if there were changes in physiological measures associated with the IVR experience (five times every session: pre-experience, 5 min, 10 min, 15 min, and post-experience).

A Student test for paired samples compared behavior during the intervention with Nature TreksVR or YouTube videos. No statistical analysis was performed for the responses to the subjective questionnaire, as all the participants assessed the experience with the best possible score in all the sessions.

## 3. Results

**Figure 2:**
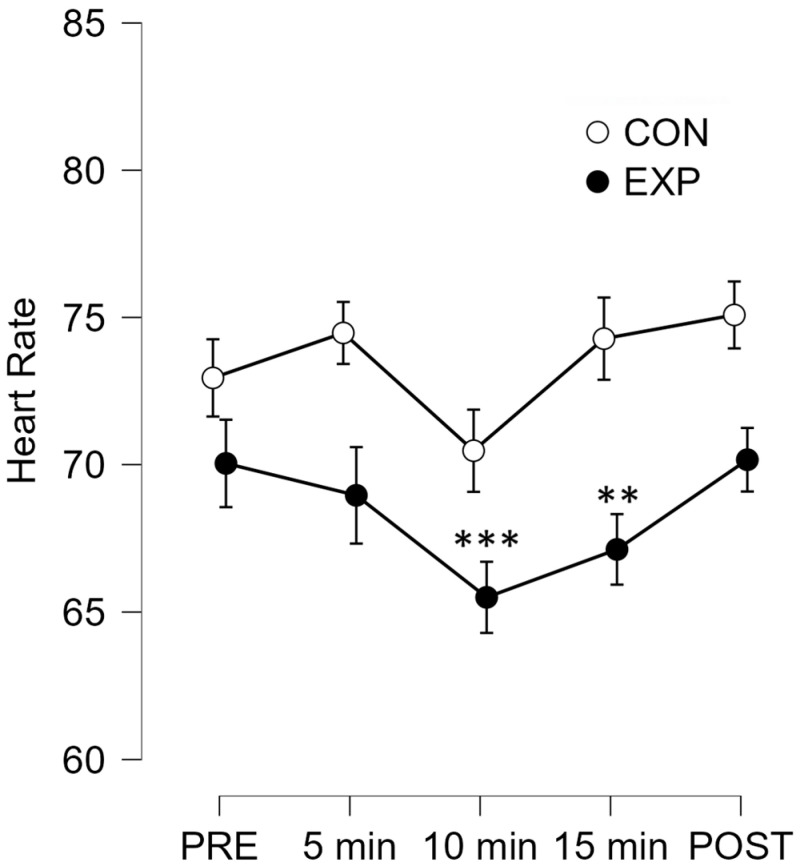
Mean heart rate (HR) values across five measurement time points (PRE, 5 min, 10 min, 15 min, and POST) for both the experimental and control conditions. The Experimental group exhibited a significant reduction in HR at the 10 min (*p* = 0.001) and 15 min (*p* = 0.01) time points compared to baseline (PRE). No significant differences were observed in the Control group across time points. Error bars represents standard errors. ** *p* < 0.05; *** *p* < 0.01.

A repeated measures ANOVA was conducted to examine changes in heart rate (HR) across five time points within each session and to assess whether this pattern differed between the Experimental and Control groups. Mauchly’s test confirmed sphericity, and Levene’s test indicated homogeneity of variances across measurement points. The analysis revealed a significant main effect of Time (F(4,56) = 5.567, *p* = 0.002, η^2^ = 0.03), indicating that HR changed over the course of the sessions. However, the Time × Group interaction was not significant (F(4,56) = 1.189, *p* = 0.326, η^2^ = 0.006), suggesting that overall HR trends did not differ significantly between conditions. Given the crossover design—where each participant experienced both conditions—it was methodologically appropriate to analyze each condition separately. This approach accounts for intra-individual variability and allows detection of condition-specific effects. In the Control group, HR remained stable throughout the session (F(4,28) = 1.252, *p* = 0.312). In contrast, in the Experimental group, there was a significant effect of Time (F(4,28) = 7.117, *p* < 0.001, η^2^ = 0.504), prompting post hoc comparisons. Holm-adjusted pairwise tests showed that HR significantly decreased at 10 and 15 min compared to the baseline (PRE) (t = 4.507, *p* = 0.001; t = 3.605, *p* = 0.01, respectively), and was still significantly lower at 10 min compared to POST (t = 3.756, *p* = 0.007). A marginal difference was also found between POST and 15 min (t = 2.854, *p* = 0.056). Figure 2 illustrates these HR trends across the five measurement points in both conditions.

We used a repeated measures ANOVA to evaluate the effects of Time (PRE vs. POST), Group (Experimental vs. Control), and their interaction on PANAS scores. Levene’s test confirmed homogeneity of variances at both PRE (*p* = 0.716) and POST (*p* = 0.387). Since only two time points were analyzed, sphericity testing was not applicable.

The analysis showed no significant main effect of Time (F(1,13) = 0.008, *p* = 0.932), indicating that overall PANAS scores did not change significantly from PRE to POST across both groups. There was also no significant main effect of Group (F(1,13) = 0.002, *p* = 0.961), meaning that PANAS scores were similar between the Experimental and Control groups. However, the interaction between Time and Group approached significance (F(1,13) = 4.497, *p* = 0.054, η^2^ = 0.062), suggesting a possible difference in how scores changed over time depending on the condition. When we analyzed each group separately, we found no significant change in the Control group (F(1,7) = 1.667, *p* = 0.238), and likewise, no significant change in the Experimental group (F(1,6) = 0.102, *p* = 0.102, η^2^ = 0.382). However, the effect size in the Experimental group indicates a moderate trend that may be meaningful with a larger sample.

**Figure 3:**
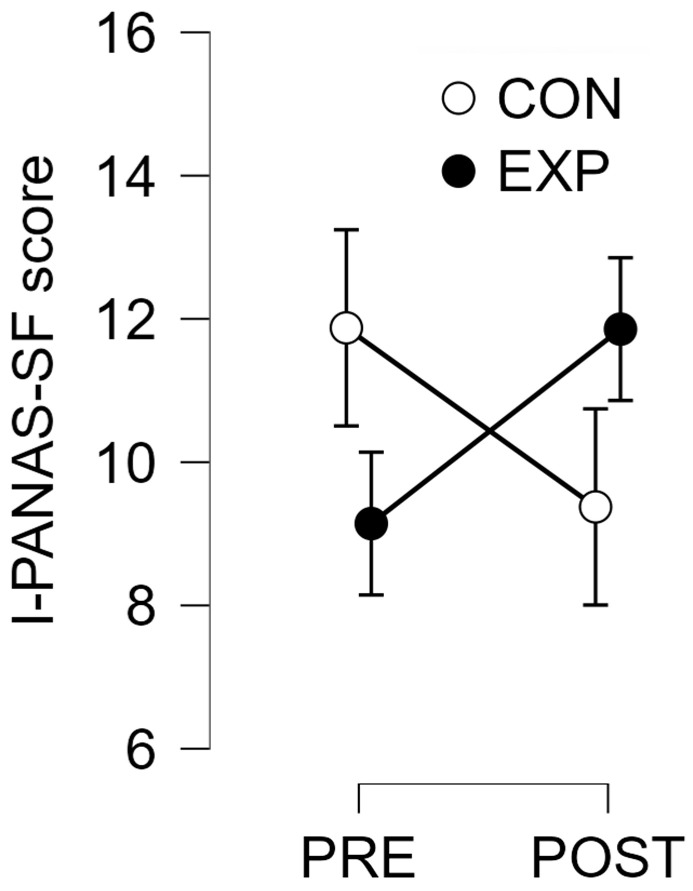
Mean PANAS (Positive Affect and Negative Affect Schedule) scores for the experimental and control conditions at PRE and POST intervention assessments. Although no statistically significant differences were observed between conditions or over time, the Experimental group showed a trend toward increased PANAS scores from PRE to POST, approaching the minimal clinically important difference (MCID). Error bars represent standard error.

To better understand the potential clinical relevance of the PANAS results, we calculated the minimal clinically important difference (MCID) using the 0.5 standard deviation method based on PRE-intervention PANAS scores. For the Experimental group, the MCID was 2.878, while the observed change from PRE to POST was +2.714—slightly below the threshold for clinical significance. In the Control group, the MCID was 2.528, and the observed change was −2.5, which also fell short of clinical significance. Figure 3 shows a comparison of I-PANAS-SF scores at PRE and POST for both groups.

We also performed repeated measures ANOVAs on the state and trait subscales of the reduced State–Trait Anxiety Inventory (STAIr). For both dimensions, we found no significant main effect of Time (State: F(1,13) = 1.156, *p* = 0.302; Trait: F(1,13) = 1.384, *p* = 0.260), nor of Group (State: F(1,13) = 0.095, *p* = 0.762; Trait: F(1,13) = 0.245, *p* = 0.629). There were also no significant Time × Group interactions (State: F(1,13) = 1.156, *p* = 0.302; Trait: F(1,13) = 0.025, *p* = 0.876).

**Figure 4:**
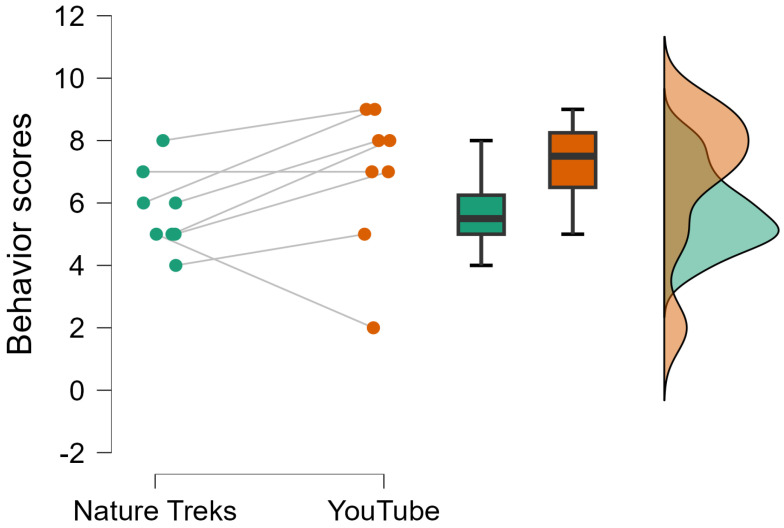
Raincloud plot showing the distribution of observed behavioral responses during the interventions, comparing the Nature Treks VR condition with the YouTube videos condition. The plot illustrates individual data points, the probability density of the distribution, and boxplots representing the median and interquartile ranges for each condition. Participants exhibited higher behavioral response scores during the YouTube condition compared to the Nature Treks VR condition, although this difference did not reach statistical significance.

We analyzed behavioral responses by comparing the average scores recorded during the Nature Treks VR and YouTube video conditions across all sessions. The Shapiro–Wilk test confirmed that the data followed a normal distribution (W = 0.861, *p* = 0.123). A paired-samples *t*-test was conducted with the directional hypothesis that responses would be lower during the Nature Treks VR sessions. The analysis revealed a trend toward significance, with participants showing higher behavioral scores during the YouTube condition compared to Nature Treks VR (t(7) = 1.624, *p* = 0.074). Figure 4 illustrates the distribution of behavioral responses across both conditions.

Regarding the subjective response questionnaire, all participants consistently gave the maximum score of 4 points in every session, indicating uniformly positive emotional reactions to the Nature Treks VR intervention. They consistently reported feeling excited and calm, and none reported feelings of upset or anxiety. Additionally, all participants confirmed the perceived realism of the virtual scenarios in every session.

## 4. Discussion

The present study aimed to evaluate the physiological, emotional, behavioral, and subjective responses of individuals with dementia during exposure to immersive virtual reality (VR) environments compared to conventional digital activities. The results provide partial support for the hypothesis that immersive VR experiences elicit beneficial effects, particularly in terms of physiological relaxation and positive subjective evaluations, although changes in self-reported emotional states and anxiety did not reach statistical or clinical significance.

Regarding heart rate (HR), the analysis revealed a significant main effect of time, with more detailed analysis showing that the VR condition led to a progressive reduction in HR during the sessions. Specifically, HR significantly decreased at the 10 and 15 min points compared to baseline, suggesting a calming autonomic response associated with exposure to VR natural environments. This reduction in HR was not observed in the control condition, where heart rates remained stable throughout. These findings are in line with previous research demonstrating the relaxing effects of virtual nature exposure and its potential to reduce physiological arousal [13]. The observed decrease in heart rate during immersive VR exposure may reflect an activation of parasympathetic processes indicative of autonomic relaxation—a response of particular relevance in older adults and individuals with dementia, given their vulnerability to stress-related physiological dysregulation. Autonomic nervous system (ANS) dysfunction is a well-documented feature of aging and dementia, contributing to impaired cardiovascular reactivity, diminished stress resilience, and reduced functional autonomy [14]. In patients with dementia, studies have shown impaired heart rate responses, reflecting ANS deterioration [15]. Against this backdrop, the physiological effects of immersive VR assume clinical relevance. The heart rate reductions observed in our study are consistent with prior research showing the capacity of VR interventions to modulate autonomic activity. For example, Mazgelytė et al. [16] reported that a single session of VR-assisted respiratory biofeedback led to significant decreases in heart rate, respiratory rate, and skin conductance, alongside improvements in mood and reductions in cortisol levels, confirming a systemic autonomic calming response. Furthermore, Rault et al. [17] observed anxiolytic effects following repeated sessions of VR relaxation in individuals with schizophrenia, highlighting the potential of IVR to serve as a transdiagnostic tool for stress regulation. In the context of dementia care, such physiological markers of relaxation may translate into practical benefits, such as reduced agitation or improved emotional regulation, especially when non-pharmacological approaches are prioritized. Supporting this, Kim and Kim [18] found that older adults aged over 70 reported marked improvements in subjective mental health, including greater relaxation, happiness, and reduced stress, following exposure to immersive natural environments via IVR. Similarly, Veling et al. [19] observed that immersive VR-based relaxation (VRelax), using 360° nature videos, was significantly more effective than standard relaxation exercises in reducing momentary anxiety and sadness in patients with psychiatric disorders. Taken together, our findings, supported by growing evidence in the literature, suggest that IVR may serve not only as a source of sensory engagement but also as a viable therapeutic strategy to promote physiological calm and enhance emotional well-being in older adults living with dementia.

In contrast, affective responses measured by the I-PANAS-SF scale did not show significant changes over time or between groups. Nonetheless, the interaction between time and group approached significance, and the observed change in I-PANAS-SF scores for the experimental group was very close to the calculated minimal clinically important difference (MCID). Specifically, while the MCID was calculated at 2.878, the observed improvement in the experimental condition was +2.714. Although this change did not strictly meet the threshold for clinical significance, its proximity to the MCID suggests a potentially promising trend that may reach meaningful levels with a larger sample or extended intervention periods. In contrast, the decrease observed in the control group did not meet the MCID threshold and may reflect random variation rather than a true effect. It is worth noting that in our study, I-PANAS-SF scores were obtained during the week preceding and following the intervention period, rather than immediately after each VR exposure. This delayed measurement may have limited the sensitivity to detect short-term mood enhancements. Supporting this interpretation, Chan et al. [20] reported significant improvements in positive and negative affect immediately after VR sessions among community-dwelling older adults.

The analysis of state and trait anxiety scores using the reduced STAIr did not reveal any significant effects, indicating no measurable changes in perceived anxiety levels as a result of the interventions. This could be due to several factors, including limited sensitivity of the tool in this population, the brief duration of exposure, or the relatively stable nature of trait anxiety. Another important consideration is the timing of the assessments. In our study, anxiety was measured during the week prior and the week after the intervention period, potentially missing the immediate anxiolytic effects of the VR sessions. Supporting this hypothesis, Niki et al. [21] demonstrated that immersive VR reminiscence significantly reduced anxiety in participants immediately following each exposure, with reductions exceeding the minimal clinically important difference for the STAI. Additionally, participants in their trial expressed a clear preference for live-action imagery over computer-generated scenes, suggesting that higher realism may enhance emotional engagement and therapeutic efficacy—a finding that aligns with our strategy of employing 360° photorealistic environments tailored to participant preferences.

Behavioral observations during the sessions indicated a trend toward higher behavioral response scores during the YouTube condition compared to the Nature Treks VR intervention. This suggests that participants were more responsive and engaged during the YouTube sessions, potentially due to the fact that these videos were directly related to their personal interests and were frequently requested by the participants themselves. Although the difference in behavioral responses did not reach statistical significance, the increased responsiveness to the YouTube condition—when compared to the inherently positive and calming effects observed during the Nature Treks VR exposure—may highlight the potential benefits of incorporating emotionally meaningful and personally relevant content into intervention programs. Although the videos were not typically autobiographical or personally familiar, their selection was driven by the participants’ own interests—such as iconic landmarks, concerts, or culturally significant places—which likely enhanced attentional engagement through increased relevance and perceived agency. This personalization, even in the absence of direct familiarity, may have acted as a moderating factor in behavioral responsiveness and should be considered in future studies comparing different types of immersive content. This finding is consistent with previous research emphasizing the importance of meaningful activities in fostering a sense of enjoyment and connection in individuals with dementia [22]. Tailoring digital activities to align with individual preferences could foster greater engagement and emotional connection, thereby enhancing the overall therapeutic value of such interventions.

Of particular note are the findings from the subjective response questionnaire, which demonstrated uniformly positive evaluations of the VR experience. All participants consistently reported feeling excited and calm, without experiencing feelings of upset or anxiety, and unanimously rated the virtual scenarios as realistic. These consistent responses point to a strong acceptance and positive perception of the VR intervention among participants. Nevertheless, it is important to consider the potential influence of social desirability bias, particularly given the vulnerable population involved. Participants may have provided consistently positive responses, at least in part, due to a desire to please the researcher or as a consequence of limited insight into their emotional state, which is not uncommon in individuals with cognitive impairments. This possibility underscores the importance of triangulating self-report measures with objective behavioral and physiological data. Future studies could consider methods to reduce social desirability bias, such as implementing anonymous or confidential self-report procedures, as recommended by Setiawati et al. [23], to enhance the validity of subjective feedback. We also plan to adopt a simplified three-point Likert scale to capture more nuanced emotional responses while preserving clarity and accessibility for individuals with cognitive impairments.

Regarding the feasibility of IVR integration into routine dementia care, recent studies offer encouraging evidence. Kim et al. [6] showed that a five-week nature-based IVR program improved emotional states and quality of life in individuals with Alzheimer’s disease, despite minor usability challenges. Likewise, a scoping review by Rose et al. [24] highlighted that semi- and fully immersive VR systems are generally well tolerated and provide valuable stimulation, particularly in early to moderate dementia stages. Complementing these findings, a recent systematic review and meta-analysis by Wen et al. [25] concluded that VR-based nursing interventions significantly improve cognitive function, quality of life, activities of daily living, and reduce negative emotions in patients with dementia. These results collectively support the practical implementation of IVR as a non-pharmacological strategy to promote emotional resilience and well-being in dementia care settings.

Finally, our findings align with the conclusions of the recent scoping review by Buele et al. [5], which reported that immersive VR interventions show positive effects on emotional well-being and neuropsychiatric symptoms in people with dementia, including reductions in apathy and depression. Although the review identified mixed results regarding cognitive improvements, it emphasized the therapeutic value of VR in enhancing emotional engagement and subjective well-being. In line with these observations, our study found physiological and self-reported indicators of emotional benefit following exposure to immersive, naturalistic VR environments, despite the absence of significant cognitive or anxiety-related changes. These results further support the use of IVR as a viable, non-pharmacological strategy to promote emotional health in dementia care, even if cognitive outcomes remain more variable and require further investigation.

### Limitations and Future Directions

A primary limitation of this study is its small sample size, which restricts statistical power and limits the generalizability of the findings. Future studies should include larger and more diverse samples to confirm these preliminary results. Extending the intervention period may also allow for the detection of longer-term effects on emotional well-being. It is also worth noting that the variability in behavioral responses across participants likely reflects individual differences in expressiveness and baseline engagement, which are common in this population. Regarding physiological outcomes, heart rate (HR) was the only autonomic marker assessed. While HR is a widely accepted and accessible indicator of autonomic arousal, it does not capture the full complexity of physiological regulation. Future studies could benefit from including additional measures such as heart rate variability, skin conductance, or respiratory patterns to provide a more nuanced understanding of the physiological effects of immersive VR. Furthermore, the use of complementary assessment tools, such as proxy reports from caregivers or behavioral observation scales tailored for dementia populations, could provide a more comprehensive evaluation of the intervention’s impact.

## 5. Conclusions

This study provides preliminary evidence supporting the feasibility, acceptability, and potential benefits of IVR interventions for individuals with mild dementia. Participants responded consistently positively to the IVR sessions, which included both nature-based environments and 360° videos of participant-selected scenarios, demonstrating high tolerability and emotional engagement. The significant reductions in heart rate observed during IVR exposure suggest a calming autonomic effect, aligning with broader findings on the psychophysiological benefits of immersive digital environments. Although changes in affective and anxiety-related self-report measures did not reach statistical significance, the PANAS scores approached both the threshold for statistical interaction and the minimal clinically important difference, indicating a promising trend. The personalized content may have played a role in enhancing the emotional salience and behavioral engagement of participants. Taken together, these findings support the use of IVR as a safe, engaging, and potentially effective non-pharmacological strategy for emotional regulation and well-being in dementia care. Future research should build on these results by increasing sample sizes, extending the intervention duration, and incorporating complementary assessment methods—such as caregiver-reported outcomes or simplified Likert scales—to capture more subtle changes in emotional response. With continued refinement, IVR may represent a scalable tool to be integrated into daily care routines for individuals with dementia.

## Figures and Tables

**Table 1:** Participants’ characteristics.

Participant	Sex	Age	CDR	GDS	Education	Occupation
CE	Female	78	1	4	Primary school	Dressmaker
CC	Female	89	1	4	Primary school	Cleaner
RM	Female	84	2	5	Primary school	Chambermaid
MG	Female	77	2	5	University	Teacher
MA	Male	83	1	3	Primary School	Driver
JA	Male	75	0.5	3	University	Architect
JM	Male	79	1	3	Primary school	Dealer
PS	Male	84	2	5	Vocational studies	Restorer of monuments

**Table 2:** Instruments used.

Psychological and Physiological Dimensions	Instrument
Level of dementia	GDS, CDR
Anxiety	STAIr
Affect	I-PANAS-SF
Heart rate	Smart Watch

## Data Availability

The original data presented in the study are openly available in FigShare at https://figshare.com/articles/dataset/ReVital_dataset_xlsx/28602026 (accessed on 15 March 2025).
